# Supplementary figures and images for: Muscle thickness and inflammation during a 50km ultramarathon in recreational runners
Source: PLoS One. 2022 Sep 1;17(9):e0273510. doi: 10.1371/journal.pone.0273510 (PMC9436055; doi:10.1371/journal.pone.0273510)

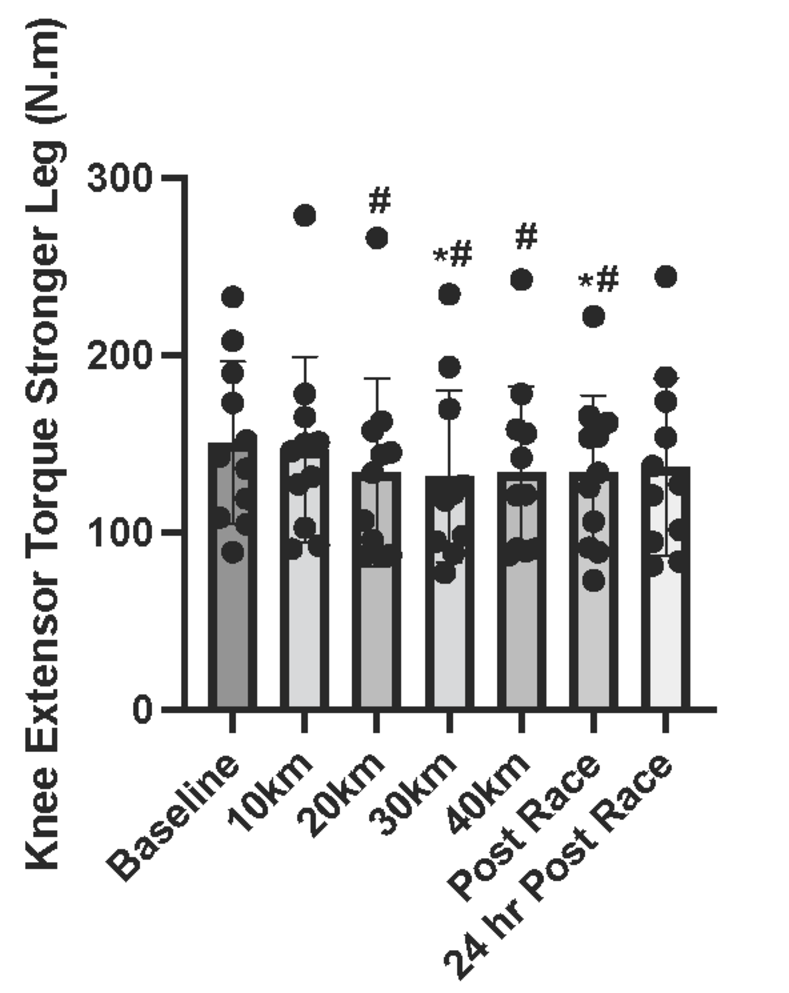

Supplement: S1 Fig — Knee extensor muscle torque at baseline, 10k, 20k, 30k, 40k, post-race, and 24 hr post-race. *indicates statistically significant from baseline, # indicates statistically significant from 10k (P ≤ 0.05). Data are reported in means ± SD. (TIF) [file pone.0273510.s001.tif]
