# Supplementary material for: Muscle thickness and inflammation during a 50km ultramarathon in recreational runners
Source: PLoS One. 2022 Sep 1;17(9):e0273510. doi: 10.1371/journal.pone.0273510 (PMC9436055; doi:10.1371/journal.pone.0273510)
Supplement: Attachment — Submitted filename: Response to Reviewers.docx [file pone.0273510.s002.docx]

We thank the reviewers for their thoughtful feedback on our manuscript. We have made substantial changes based in the reviewer’s recommendations and feel this revised manuscript is significantly improved. Specifically, as suggested by both reviewers, we have added clarity to the interpretation of ΔMT and provided alternative explanations for our findings. Detailed responses to each comment are provided below. For ease of review, we have included the **reviewer’s original comment in bold** and our response directly below. Changes made within the manuscript are highlighted in red with the line number indicated.

**Reviewer #1: 

The biggest problem of this manuscript is that all the tables are missing. This reviewer cannot find any tables in the submitted file.**

A per journal requirements, tables have been re-located directly after the paragraph in which they are first cited.

**One of the major limitations of this manuscript is the weak introduction section, which makes the novelty and relevance of the study weak. For example, what are the functions of IL-6, sIL-6R, calprotectin? Why are they important to measure in this study? What is the difference between IL-6 and sIL-6R, and why are they both needed to measure instead of either one? What is the rationale of measuring muscle thickness? What does the ΔMT mean vs. MT physiologically? What is the rationale of analyzing ΔMT vs. just MT data? How is MT/ ΔMT related to muscle swelling or inflammation?**

We have significantly revised the introduction to improve clarity. In keeping with journal guidelines regarding the length of the introduction, further details about the respective roles of each inflammatory factor measured and how we interpret these factors affecting the other results in this study have been included in the discussion.

We have also clarified the novelty and importance of including both the contracted MT and ∆ MT measures as a more comprehensive picture of changes in muscle morphology beyond just resting measures which have been the major focus of previous studies.

Line 76

“Ultramarathon running, specifically, has been found to elicit large increases in IL-6 (3–5) which has numerous physiological roles. When bound to its soluble receptor (sIL-6R), IL-6 acts through a trans-signaling pathway to promote cellular apoptosis (6). Other inflammatory factors such as tumor necrosis factor (TNF)-α (3, 7–9), and calprotectin (10, 11) are elevated after prolonged running and are involved in the activation and breakdown of damaged cells (10, 12).”

Line 85

“Further, inclusion of MT measures during muscle contraction may provide more insight into changes in muscle morphology post-exercise as it may reflect shifts in intramuscular fluid pressure (17), or altered muscle recruitment, especially when used in conjunction with measures of muscle strength (18). Therefore, reporting MT under both resting and contracted conditions as well as ∆ MT to demonstrate changes relative to resting conditions provides a more comprehensive picture of acute changes in muscle morphology in response to ultramarathon running.” **Detailed comments:
1. Lines 259,261, 266: Where are the Table 1 & Table 2?**

A per journal requirements, tables have been re-located directly after the paragraph in which they are first cited.

**2. Line 265: “ were not considered elite.”
- What is the definition of eliteness?**

We define elite status as determined by measured VO2max values in comparison to several well-established reports of VO2max in competitive endurance athletes across various ages (Heath et al. JAP 1981; Robinson et al., JAP 1976; Foster et al. Eur J Appl Physiol and Occup Physiol 1978; Bergh et al. Med Sci Sports 1978). Our average VO2max value was ~50 ml/kg/min and values of 70 ml/kg/min or higher are frequently reported in elite endurance athletes. We have added these references to the manuscript on line 276.

**3. Line 273: “non-significant increase of 55% from baseline to post-race (P= 0.217, d = 0.5)” .
- If there are no significant changes, it should not be described as non-significant changes.**

This has been corrected.

**4. Line 353: “In our observation, post-race inflammation may contribute to delayed alterations in muscle recruitment exhibited as greater ΔMT noted 24-hrs post-race”
- How is ΔMT related to muscle recruitment? This should be explained in the introduction section. It is confusing that greater ΔMT indicates muscle recruitment alterations.**

Clarification on contracted and ΔMT have been added to the introduction as indicated above.

Inflammation immediately post-race (supported by elevations in several inflammatory cytokines) may influence neuromuscular recruitment (Millet et al. 2018 APNM) and alter motor unit responsiveness leading to less efficient motor unit contraction (greater recruitment to perform the same task). Indeed, we found that contracted MT exhibited a delayed elevation at 24-hrs post-race that, while not statistically significant, was substantially greater than immediately post-race and in the absence of resting MT changes, appears to be a potential contributor to our observed changes in ΔMT. The influence of inflammation on muscle recruitment has been added to the introduction:

In response to feedback from both reviewers, we have elaborated on this theory in the discussion and also posed an alternative explanation to our observed changes in ΔMT.

Line 392

“In our observation, inflammation exhibited post-race may contribute to altered skeletal muscle recruitment patterns at 24-hrs post-race such that over-recruitment (32), as evidenced by greater contracted MT, is required to produce the same force when asked to maximally contract the quadricep (12, 32). This would exhibit as greater ∆ MT 24-hrs post-race. Future studies including EMG while assessing MT in the contracted state are needed to confirm this hypothesis. Alternatively, while our results suggest a potential temporal effect of inflammation on elevations in MT 24-hrs post-race, we cannot exclude other explanations. For instance, other studies have documented associations between elevated intramuscular fluid pressure and delayed onset of muscle soreness following repeated eccentric activity (15). This may be more evident in the muscle during the contracted state, causing the increases in ΔMT observed 24-hrs post-race. While the exact cause of ΔMT is not well understood, our findings substantiate the inclusion of MT during the contracted state as it provides insight into changes in muscle morphology post-race that were not detected in the resting state. However, as this is one of the first studies to utilize ultrasound in both resting and contracted conditions, further research is needed to fully understand what is causing alterations in MT during contraction.”

**5. Line 360-361: “the rectus femoris has been reported to have the least amount of change of the quadriceps muscles during races”
- Then why chose it as the muscle group for the measurements?**

Since the rectus femoris is superficial we selected this muscle because we were able to quickly acquire images in the field with a single ultrasound probe across various body sizes in both the resting and contracted state.

Originally, we stated “the rectus femoris has been reported to have the least amount of change of the quadriceps muscles during races with heavy eccentric loads, as it sustains the least stimulation during downhill running (32) This suggests that for studies with greater elevation changes, other quadriceps muscles should be included in the assessment.” Our original intent was to discuss our findings in a race with little elevation change, in comparison to many of the previous studies that have examined runners after higher levels of elevation change. As noted in paragraph 2 of the discussion, line 366, the race performed for this study had only 762 meters of elevation change over ~31 miles with no net loss, making the eccentric loads sustained in this study minimal. While the rectus femoris is appropriate for our study that had minimal elevation change, if races with greater elevation change, and therefore, greater eccentric loading are to be included, it would be important to include other quadriceps muscles as well.

To clarify our original statement, we now write:

(Line 413)

“Thus, while the rectus femoris is appropriate to assess in this race due to minimal elevation change, other quadriceps muscles should be included for studies in which races are performed with great elevation loss.”

**6. Lines 401-402: “This study was designed to determine the practicality of using ultrasound as a field-based measure..”
- The results don’t support this. This reviewer couldn’t tell that the data showed the practicality of using ultrasound as a measure of the inflammation during the race. Needs more explanation/justification.**

We appreciate the question and have rephrased this sentence to better represent the point that, while ultrasound can be used to assess muscle swelling, as a field-based measure it comes with some limitations.

Line 461

“While our data support the use of ultrasound as a field-based measure of muscle morphology, this study did come with some limitations.”

**Reviewer #2: 

1. The authors do not comment on the development of peripheral or central fatigue and how that might have impacted inflammation, strength, and performance. The authors, rightly in my mind, discuss the idea of muscle damage that may have occurred during the race and how this could have impacted MT and strength. However, the loss of force production during the race may simply have been due to fatigue. Is there a relationship between fatigue and inflammation? If so, how might this have impacted the results?**

We agree that commenting on peripheral and central fatigue is a valuable addition to this manuscript and thank this reviewer for the opportunity to include this in our discussion. We have added content to the discussion regarding the effects of central and peripheral fatigue as well as the relationship between inflammation and fatigue.

Line 448

“Both central and peripheral fatigue can explain decrements in force production following an ultramarathon (14, 39) and have been shown to persist for well over 30 minutes post-exercise in endurance events lasting several hours (34). While not directly assessed in this study, these past findings suggest that it is likely that both central (motor unit recruitment) and peripheral fatigue (metabolite buildup and/or impaired excitation-contraction coupling) contribute to reduced post-race knee extensor torque and may also be influenced by inflammation. Specifically, inflammation can inhibit neural drive by interfering with afferent feedback from exercising muscles (14, 40).” **2. I appreciate the effort of the authors to conduct field-based research given its difficulties. I am curious as to why participants were only retested at 24 hours post? Muscle damage often manifests for days to week after eccentric exercise with inflammation due to muscle damage often peaking 7-10 days after the insult. Given that force production had essentially returned to baseline levels and that no measure of muscle soreness was collected it is very difficult to conclude that appreciable damage actually occurred--indeed the fact that force was recovered 24hr post strongly suggests that decrements during exercise were simply due to fatigue. Thus any change in blood markers of inflammation or MT may or may not be due to damage.**

We thank the reviewer for this comment. Not including measures beyond 24-hrs post-exercise is a limitation in our study as noted in line 469. Specifically, many of our participants were not local and were therefore unable to come in for testing on a weekday morning due to work obligations. We agree with this reviewer that, given the timeframe in which we were able to collect post-race data, we cannot conclude that these changes in MT or inflammation are due specifically to muscle damage. Rather, we have selected the terms inflammation and swelling which we feel more accurately represent the findings in this study. We have revisited the manuscript to remove conclusions that imply that our results reflect muscle damage and have added the following sentence to address this point

line 381:

“Importantly, muscle damage and associated peaks in CK are most apparent several days after the initiating event. Thus, we were not able to distinguish whether the findings in our study are due to muscle damage or other fatigue-related factors.”

**3. Do the authors feel that changes in inflammatory markers during exercise could simply have been the "normal" response and may actually not play any role in changes in MT, force, or performance?**

We have included this possibility in our limitations section.

Line 473

“Finally, while there is a documented relationship between inflammation and muscle function (11, 12, 25), we cannot mechanistically conclude that the inflammatory factors we assessed were involved in reduced knee extensor torque or alterations in ∆ MT.” **4. I know the sample size is small, but reporting correlations between changes in MT, force, and blood markers could be help for beginning to understand the relationship among them.**

Due to difficulty obtaining full sets of blood samples from participants, our ability to perform correlations between inflammatory factors, MT and force production was limited. To not mislead our readers, we have opted to exclude these correlations from the study. We agree this would be great future direction and have added it into the discussion.

Line 476

“Correlations between the measured inflammatory factors and functional outcomes were not possible due to limited blood samples, but should be included in future studies to better explain these relationships”

**5. Are there alternative explanations for the change in MT during exercise beyond inflammation? Changes in intramuscular pressure as well as changes in oncotic pressure due to metabolism could easily function to drive water out of blood and the interstitial space and into skeletal muscle fibers. I feel this should be discussed. The fact that it remains elevated 24 hours later is suggestive of a longer lasting effect, which could be from inflammation.**

We thank this reviewer for the added insight into potential increases in ΔMT observed in our study. We have added the following statement to address this in the discussion section.

Line 397:

“Alternatively, while our results suggest a potential temporal effect of inflammation on elevations in MT 24-hrs post-race, we cannot exclude other explanations. For instance, other studies have documented associations between elevated intramuscular fluid pressure and delayed onset of muscle soreness following repeated eccentric activity (15). This may be more evident in the muscle during the contracted state, causing the increases in ΔMT observed 24-hrs post-race. While the exact cause of ΔMT is not well understood, our findings substantiate the inclusion of MT during the contracted state as it provides insight into changes in muscle morphology post-race that were not detected in the resting state. However, as this is one of the first studies to utilize ultrasound in both resting and contracted conditions, further research is needed to fully understand what is causing alterations in MT during contraction.” **6. I think the idea that understanding how acute changes in MT and inflammation, which could be tracked by ultrasound, could lead to increased risk of musculoskeletal injury needs to be further developed. Is there any evidence to support such a suggestion?**

This is the first of several studies in our lab that use ultrasound imaging to detect differences in muscle morphology in response to a variety of muscle stimulation/exercise protocols. Long-term, we hope that by documenting changes in response to these training and competition-based stimuli may be able to help predict injury risk. However, in line with what this review has noted, the connection is not yet clear. We have edited this section in the discussion to better reflect our plans for the future.

Line 480

“In this study, we document changes in MT in response to prolonged, submaximal muscle contractions. Future studies will investigate MT in response to other stimuli with varying degrees of duration and intensity with the long-term goal of informing mitigation strategies to minimize musculoskeletal damage that may impair performance.” **Specific Comments
1. p13, ln286 - please do not refer to non-significant results as a "tendency"**

This has been removed.

**2. p16, ln 353 - how can post race inflammation be related to changes in recruitment as indicated by MT measures? This seems very tenuous at best.**

While not believed to be the only explanation, there is some evidence that inflammation can interfere with afferent feedback from exercising muscle and alter motor unit responsiveness (Millett et al. APNM 2018). We have added this as a potential explanation. Additionally, as indicated above, we have proposed another potential explanation for the observed changed in MT.

Line 392

“In our observation, inflammation exhibited post-race may contribute to altered skeletal muscle recruitment patterns at 24-hrs post-race such that over-recruitment (32), as evidenced by greater contracted MT, is required to produce the same force when asked to maximally contract the quadricep (12, 32). This would exhibit as greater ∆ MT 24-hrs post-race. Future studies including EMG while assessing MT in the contracted state are needed to confirm this hypothesis.”

**3. p19, ln 424 - I would caution the authors about suggesting muscle damage is part of a typical remodeling response. Damage in a mechanical event, likely due to eccentric exercise. Following the damage, there will likely be repair and perhaps remodeling that will occur.**

This statement has been removed.
