# Supplementary material for: Muscle thickness and inflammation during a 50km ultramarathon in recreational runners
Source: PLoS One. 2022 Sep 1;17(9):e0273510. doi: 10.1371/journal.pone.0273510 (PMC9436055; doi:10.1371/journal.pone.0273510)
Supplement: Attachment — Submitted filename: Response to Reviewers.docx [file pone.0273510.s003.docx]

We thank this reviewer for their thoughtful feedback on our manuscript. In this revisions, we have clarified the rationale for the inflammatory factors selected in this study. Further, we expanded the discussion of our findings related to inflammation and our major outcome measures. Detailed responses to each comment are provided below. For ease of review, we have included the **reviewer’s original comment in bold** and our response directly below. Changes made within the manuscript are highlighted in red with the line number indicated.

1) **It is still not very clear why the authors chose IL-6, sIL-6, TNF-alpha, calprotectin as the inflammatory markers to measure. For example, why didn’t the author measure other commonly used inflammatory markers such as CRP, IL-1B?**

We appreciate this reviewer’s interest in the other inflammatory markers that may be affected by ultramarathon running. A comprehensive panel inflammatory factors was not possible for this study so we selected factors with established roles in systemic inflammation that have been previously found to be influenced by prolonged exercise and may affect muscle function. The roles of the selected cytokines are now further defined in the introduction. Additionally, we have included a statement in the limitations to address not including a broader panel of inflammatory factors.

Line 76: Ultramarathon running, specifically, has been found to elicit large increases in IL-6 [3–5]. Among its numerous physiological roles, IL-6 may limit motor activity as a protective mechanism in response to fatigue from prolonged or intense exercise [6,7]. Further, when bound to its soluble receptor (sIL-6R), IL-6 acts to promote cellular apoptosis [8] and the inflammatory properties may be amplified [7]. Other inflammatory factors such as tumor necrosis factor (TNF)-α [3,9–11], and calprotectin [12,13] are elevated after prolonged running and are involved in the activation and breakdown of damaged cells, including skeletal muscle cells, through inflammatory signaling pathways [12,14]. Together, these factors may contribute to declines in muscle function (e.g., acute decreases in strength) [15] seen after an ultramarathon event and play a role in subsequent recovery [16].

Line 487: “…future studies should expand the inflammatory profile examined and include potential anti-inflammatory cytokines for a more comprehensive understanding of the inflammatory process and associated mechanisms.”

2) **Line 77: “found to elicit large increases in IL-6 [3–5] which has numerous physiological roles”
It would be better to expand this sentence on the role(s) of IL-6, especially its roles closely related to this study.**

**Line 78: Same issue with sIL-6R. Are there insoluble IL-6Rs? What does “tran-signaling pathway”**

We have expanded the description of the role of IL-6 that are most relevant to this study. IL-6 has both membrane-bound and soluble receptors. Studies have found that the role of IL-6 is dependent on the receptor and subsequent signaling pathway that is elicited (Scheller et al., *Biochimica et Biophysica Acta-Molecular Cell Research* 2011). We selected sIL-6R as elevations in both IL-6 and sIL-6 receptor would be indicative of trans-signaling pathway and would suggest inflammation and apoptosis. This has been clarified in the introduction.

Line 76: “Ultramarathon running, specifically, has been found to elicit large increases in IL-6 [3–5]. Among its numerous physiological roles, IL-6 may limit motor activity as a protective mechanism in response to fatigue from prolonged or intense exercise [6,7]. Further, when bound to its soluble receptor (sIL-6R), IL-6 acts to promote cellular apoptosis [8] and the inflammatory properties may be amplified [7].”

**3) Lines 86&87: “into changes in muscle morphology post-exercise as it may reflect shifts in intramuscular fluid pressure [17], or altered muscle recruitment”
Please explain these further. For example, what kinds of shifts in intramuscular fluid pressure? What kinds of muscle recruitment alterations can be indicated by the MT?**

This has been explained in more detail in the introduction:

Line 89: For instance, contracted MT may emphasize intracellular swelling due to ions released from cytoskeletal breakdown after repetitive muscle contractions and resulting in shifts in osmotic pressure gradients [19]. Further, contracted MT may reflect altered muscle recruitment (failure to fully recruit or over-recruitment in response to a standard task).

**4) As one of the main outcome measures, CK was not mentioned at all in the introduction section. What is the rationale of CK measurement in this study?**

While involved in the inflammatory process, CK is considered a marker of muscle damage and not inflammation and was therefore not included as a main outcome measure. Rather, CK, along with hematocrit and leukocyte, were included as secondary measures to confirm or exclude the existence of muscle damage (CK), dehydration (hematocrit), and an inflammatory response (leukocytes). These outcomes are all included in the discussion as a means of comprehensively interpreting our results. The methods have been clarified to reflect the purpose of including these outcomes.

Line 259: “As secondary outcomes, blood samples obtained pre-race, 10km, post-race, and 24 hrs post-race were also analyzed for creatine kinase (CK), hematocrit, and leukocytes (Quest Diagnostics, Baltimore, MD) to assess disruption to muscle fibers, dehydration, and an inflammatory response, respectively.”

**5) Line 98: “men with a higher VO2max (>60 ml/kg/min) compared to those with a VO2max < 55 ml/kg/min”.**
**Are these values the threshold values to define the elite runners vs. recreational runners? If so, the authors should define these terms somewhere here or in the section of “Participant selection”.**

The VO2max cutoffs that this reviewer is referencing come from a previously published article by Mooren et al. *Int J Sports Med*, 2006 and was not established by the current authors. In the Mooren et al. paper the authors do not specify the criteria they used to determine this threshold. We did not identify a specific VO2max value in our inclusion criteria. Rather, we performed VO2max tests to define subject characteristics and confirm that the values recorded are consistent with recreational runners using references from multiple previous studies provides in line 282 of our manuscript.

**6) Lines 275&276. What were the largest and smallest VO2max values of the subjects?**

The lowest recorded VO2max in our study was 43.5 ml/kg/min while the highest was 62.1 ml/kg/min. This range has been added to line 282.

**7) Overall, this reviewer still doesn’t understand how the findings demonstrate that MT measurement “may increase sensitivity to muscle changes” (Line 419). This study found that ΔMT (both absolute and relative values) was significantly increased at 24hr-post race. However, inflammatory cytokines like IL-6 were increased immediately post-race, which seemed more sensitive to the muscle changes?**

We agree that this sentence was misleading and have removed the sentence. Further interpretations of the MT findings are outlined in the response to the reviewer’s next comment below.

**8) Lines 396-406: There were significant changes in the deltaMT, but no significant changes in the MT. Any discussion on that? Does that mean future studies should use deltaMT instead of MT to analyze the muscle changes?**

We thank this reviewer for highlighting the critical findings of our study. The paragraph beginning on line 369 discusses the results for resting MT and why we suspect these findings occurred. Specifically, the elevation change in this race was low compared to other documented ultramarathons, which may result in less disruption to the cell membrane that would cause swelling in the resting state.

Line 369: “While prolonged low-load exercise protocols contribute to muscular swelling or edema [28], this was not observed as greater resting rectus femoris MT following the 50km in our study. Elevation changes, especially elevation loss, can increase muscle swelling and inflammation due to the repeat eccentric contractions that occur during a race with large elevation loss [29]. Common to ultramarathon running, many races exceed 3,000 m [3,11,22,30–32] in elevation change over the course of the race. However, by comparison, the current study’s race had a total elevation change just under 762 m with no net gain or loss.”

The next paragraph beginning on line 384 discusses the ∆ MT findings and potential reasons for these findings with a focus on the contracted state.

Line 384: “When compared with baseline, we observed a significantly higher absolute and % ∆ MT at 24hrs post-race. To our knowledge, this is the first account of MT being reported as the ∆ MT from resting to contracted conditions in this non-elite ultrarunning population. The 24-hr post-race increase in ∆ MT is consistent with other reports of delayed onset of muscle soreness [29,35] and is supported by the clinically meaningful increase in CK at 24hrs post-race.”…..

Line 398: “…The inflammatory response to exercise is highly variable and appears dependent on the extent of the muscle impairment [29]. In our observation, inflammation exhibited post-race may contribute to altered skeletal muscle recruitment patterns at 24-hrs post-race such that over-recruitment [36], as evidenced by greater contracted MT, is required to produce the same force when asked to maximally contract the quadricep [16,36]. This would exhibit as greater ∆ MT 24-hrs post-race. Future studies including EMG while assessing MT in the contracted state are needed to confirm this hypothesis. Alternatively, while our results suggest a potential temporal role of post-race inflammation on elevations in MT 24-hrs post-race, we cannot exclude other explanations. For instance, other studies have documented associations between elevated intramuscular fluid pressure and delayed onset of muscle soreness following repeated eccentric activity [19]. This may be more evident in the muscle during the contracted state, causing the increases in ΔMT observed 24-hrs post-race.”

Collectively, the above-mentioned paragraphs discuss the proposed mechanisms behind each measure, provide potential reasons for why significant differences may not be present in ∆ MT but not the resting state, and advocate for the inclusion of both resting and contracted MT measures as they provide insight into different mechanisms that can be explored using ultrasound (see line 407 as pasted below).

Line 410: “While the exact mechanisms contributing to changes in ΔMT are not well understood, our findings substantiate the inclusion of MT in both the resting and contracted state to allow for greater insight into changes in muscle morphology in response to acute exercise.”

**9) Lines 430&431: “The specific role of calprotectin in response to exercise is not completely understood”
Then why did the authors choose to measure calprotectin? Needs to be explained in the introduction.**

Calprotectin was selected due to previous reports of it being release by and subsequently acting on skeletal muscle in response to prolonged contraction and its role in the activation and breakdown of damaged cells through inflammatory signaling pathways (Mortensen et al., *J Physiol* 2008). This is described in line 81-82 of the introduction and the combined role of IL-6 and calprotectin as it relates to our findings in described in more detail in the revised discussion.

Line 81: Other inflammatory factors such as tumor necrosis factor (TNF)-α [3,9–11], and calprotectin [12,13] are elevated after prolonged running and are involved in the activation and breakdown of damaged cells, including skeletal muscle cells, through inflammatory signaling pathways [12,14]. Together, these factors may contribute to declines in muscle function (e.g., acute decreases in strength) [15] seen after an ultramarathon event and play a role in subsequent recovery [16].

Line 422: “…IL-6 and muscle contractions also promote the release of calprotectin from skeletal muscle [38] which was observed in the present study during and following the 50km race. The specific role of calprotectin in response to exercise is not completely understood, but it has been found to activate cellular pathways promoting inflammation [13,38]. Inflammation induced by elevated concentrations of IL-6 and calprotectin post-race may have a collective effect on motor recruitment patterns [7] or contraction-induced swelling [38] that exhibit as physiological phenotype (i.e. greater absolute and % ∆ MT) 24 hrs post-race.

**10) Lines 422-429: The discussion on the role of IL-6 here is very confusing. The greater ΔMT was observed at 24hr post-race when the “IL-6 levels were nearly returned back to baseline”. How does “the greater absolute and % ΔMT observed at 24 hrs post-race” support “IL-6 may aid in muscle regeneration in response to exercise through the classical IL-6 signaling pathway associated with anti-inflammatory properties [6].”? How does this suggest “a delayed inflammatory response”?**

As with many cytokines, the peak in plasma concentration often presents earlier than the physiological phenotype (i.e . ΔMT). We recognize that the term “delayed inflammatory response” may not best represent this point and have revised this sentence accordingly. Further, we have rearranged this paragraph to explain the potential role of IL-6 and calprotectin in ΔMT more clearly.

Line 426: “Inflammation induced by elevated concentrations of IL-6 and calprotectin post-race may have a collective effect on motor recruitment patterns [7] or contraction-induced swelling [38] that exhibit as physiological phenotype (i.e. greater absolute and % ∆ MT) 24 hrs post-race”

**11) Line 424: Why sIL-6R levels were not affected? Any discussion on that?**

The absence of change in sIL-6R suggests that IL-6 was acting on its membrane-bound receptor (as opposed to the soluble receptor). This suggests that IL-6 is playing a role in metabolic recovery post-exercise. This has been further clarified in the discussion.

Line 416: In the present study we found that plasma IL-6 levels increased substantially throughout the 50km while sIL-6 receptor levels were not significantly affected. Although still slightly elevated, IL-6 levels were nearly back to baseline by 24hrs post-race. IL-6 is consistently elevated in response to exercise even in the absence of muscle damage [38], while elevations in sIL-6R are variable [7]. No changes in sIL-6R suggest that IL-6 is acting primarily through its membrane-bound receptor [8]. Through this pathway, IL-6 most likely aids in fat oxidation and glucose disposal to promote recovery post-exercise [38].

**12) Lines 444-446: “The decline in muscle strength… may contribute to …observed increase in ΔMT…”. So, the changes in ΔMT were due to the muscle strength decline? Very confusing.**

We have revised this paragraph to better express the role that the strength measures play in the interpretation of our findings.

Line 441: “Both central and peripheral fatigue can explain decrements in force production following an ultramarathon [16,41] and have been shown to persist for well over 30 minutes post-exercise in endurance events lasting several hours [36]. While not directly assessed in this study, these past findings suggest that it is likely that both central (motor unit recruitment) and peripheral fatigue (metabolite buildup and/or impaired excitation-contraction coupling) contribute to reduced post-race knee extensor torque and may also be influenced by inflammation. Specifically, inflammatory factors, including IL-6 [7], can inhibit neural drive by interfering with afferent feedback from exercising muscles [16,42]. In our study, this may be exhibiting as 24 hr post-race declines in muscle strength and compensatory increases in ΔMT representing altered motor unit recruitment [7,16,42].”

**13) Line 509: “…delayed onset of muscle inflammation…”. What does this mean? Does it mean the muscle inflammatory response only occurred at a later timepoint like 24hr post-race in this study? Needs to be careful with “delayed onset” here because the acute inflammatory response obviously already initiated quickly post-race or even during the race as indicated by the increased IL-6 levels at 10km and post-race.**

This sentence has been revised to better represent the conclusions:

Line 519: “Collectively, these findings indicate the presence of some residual strength impairments and morphological changes to the rectus femoris 24-hrs following a 50km ultramarathon in recreational runners.”

**14) One of the main rationales of this study was to investigate the “trajectory of the inflammatory process in non-elite athletes..”(Line 103). It would be nice if the authors discuss whether their findings matched with those of studies in elite athletes.**

Aside from training status, several factors are involved in the release of inflammatory cytokines including duration, intensity, and elevation changes within the course. As we do not have a group of elite runners performing the same race, these various confounding variables prevent us from comparing our results to those of elite athletes in other studies. We have added this as a limitation to our discussion.

Line 464: “Findings from this study provide insight into the muscle inflammatory response to ultramarathon running in recreational runners. Differences in race duration, intensity, and elevation change do not allow for direct comparisons between our findings and those from elite runners. Future studies should include both recreational and elite runners to determine whether findings are consistent in individuals with greater fitness levels and training.”

**15) It was not clear how the findings from this study can help “develop training plans for longer events and design recovery strategies to optimize skeletal…”(Line 513). Pleaser provide more direct connection between them.**

These sentences have been revised:

Line 523: “It not uncommon for recreational ultramarathon runners to participate in stage races, back-to-back long runs, or weekly marathon and 50k distances [2]. Thus, these findings may be used by athletes, coaches, and trainers to optimize recovery strategies for skeletal muscle health, function, and inflammation when partaking in subsequent prolonged running events.”
